# The Endothelium Is Both a Target and a Barrier of HDL’s Protective Functions

**DOI:** 10.3390/cells10051041

**Published:** 2021-04-28

**Authors:** Jérôme Robert, Elena Osto, Arnold von Eckardstein

**Affiliations:** Institute of Clinical Chemistry, University of Zurich and University Hospital of Zurich, 8091 Zurich, Switzerland; Jerome.Robert@usz.ch (J.R.); Elena.Osto@usz.ch (E.O.)

**Keywords:** high-density lipoprotein, HDL, endothelial cells, transcytosis, signaling, SR-BI, S1P, endothelial lipase, ABCG1

## Abstract

The vascular endothelium serves as a barrier between the intravascular and extravascular compartments. High-density lipoproteins (HDL) have two kinds of interactions with this barrier. First, bloodborne HDL must pass the endothelium to access extravascular tissues, for example the arterial wall or the brain, to mediate cholesterol efflux from macrophages and other cells or exert other functions. To complete reverse cholesterol transport, HDL must even pass the endothelium a second time to re-enter circulation via the lymphatics. Transendothelial HDL transport is a regulated process involving scavenger receptor SR-BI, endothelial lipase, and ATP binding cassette transporters A1 and G1. Second, HDL helps to maintain the integrity of the endothelial barrier by (i) promoting junction closure as well as (ii) repair by stimulating the proliferation and migration of endothelial cells and their progenitor cells, and by preventing (iii) loss of glycocalix, (iv) apoptosis, as well as (v) transmigration of inflammatory cells. Additional vasoprotective functions of HDL include (vi) the induction of nitric oxide (NO) production and (vii) the inhibition of reactive oxygen species (ROS) production. These vasoprotective functions are exerted by the interactions of HDL particles with SR-BI as well as specific agonists carried by HDL, notably sphingosine-1-phophate (S1P), with their specific cellular counterparts, e.g., S1P receptors. Various diseases modify the protein and lipid composition and thereby the endothelial functionality of HDL. Thorough understanding of the structure–function relationships underlying the multiple interactions of HDL with endothelial cells is expected to elucidate new targets and strategies for the treatment or prevention of various diseases.

## 1. Introduction

**Figure 1:**
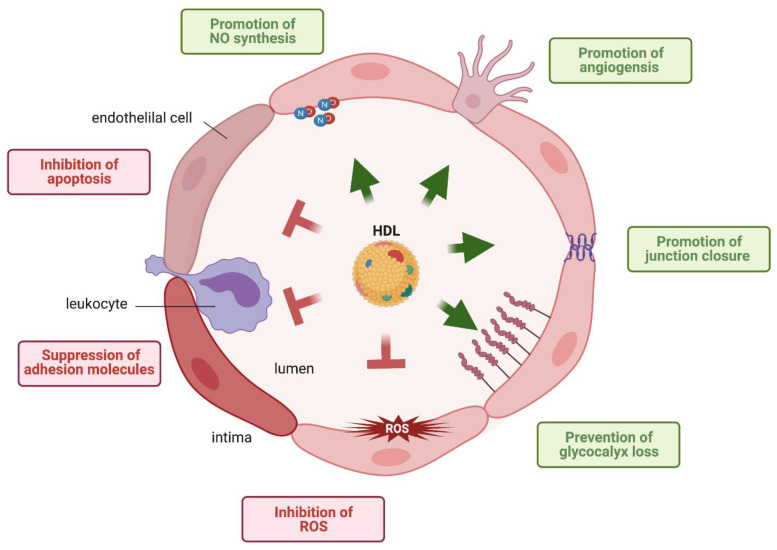
Vasoprotective functions exerted by HDL on the endothelium. HDL increases (green) vasoprotective functions and suppresses (red) harmful functions of the endothelium.

High-density lipoproteins (HDL) are best known for their role in reverse cholesterol transport (RCT), the process by which excess cholesterol is removed from cells and transported to the liver for excretion into the bile [1]. Notably, HDL is a heterogeneous mixture of lipoprotein particles that are distinguished from each other by the composition of several hundreds of different lipid and protein species [2,3,4]. Interestingly, only one-third of the identified proteins have roles in lipid metabolism, with the remaining functioning in protease inhibition, complement regulation, hemostasis and inflammation [3,5]. In particular, HDL exerts several potential vasoprotective functions on the endothelium, namely; (i) induction of endothelial nitric oxide (NO) synthase (eNOS) resulting in vascular relaxation, (ii) suppression of endothelial adhesion molecules such as vascular adhesion molecule (VCAM)1 and induced cellular adhesion molecules (ICAM1), thereby preventing leukocyte transmigration through the vascular wall or perivascular tissues, (iii) inhibition of reactive oxygen species (ROS); (iv) inhibition of apoptosis, (v) promotion of angiogenesis and endothelial repair, (iv) promotion of endothelial junction closure and (vii) prevention of glycocalix loss (Figure 1). In addition to being a target of protective HDL functions, the endothelium is also a barrier for HDL to exert its protective effects in extravascular compartments. Indeed, HDL is found in several tissues and body fluids separated from the bloodstream by selectively permeable endothelial barriers. Of note, the classical function of HDL, the induction of cholesterol efflux from macrophage foam cells in atherosclerotic plaques requires two passages through the endothelial barriers, one to enter the arterial intima and one to return into the bloodstream, probably via lymphatics [6]. Moreover, HDL-like particles are found within the brain parenchyma and cerebrospinal fluid (CSF). Most HDL particles in the brain contain apolipoprotein (apo)E as the main protein and are therefore likely of local rather than systemic origin [7]. However, some HDL contain apoA-I that is not intrinsically produced within the brain and have likely entered the brain from the systemic circulation through the endothelium of the blood–brain barrier (BBB) [8]. In this review, we first describe the transport of HDL through the endothelial barrier and the different signaling mechanisms by which HDL regulates the functions and integrity of the endothelium. Then, we discuss the alterations of these relationships between HDL and endothelial cells in different diseases. Finally, we summarize our knowledge on the interaction of HDL with the BBB. 

## 2. HDL Transport through Endothelial Cells

The endothelium is a polarized monolayer covering the luminal side of blood vessels including arteries, capillaries, veins, but also the CSF barrier and lymphatic system. The endothelial cells form a barrier restricting molecules and cells from exchanging between blood and tissues or extravascular space [9]. Three types of intercellular junctions; namely gap, adherens and tight junctions connect adjacent endothelial cells. The organization, the number and the composition of these junctions underlie the differences of vessel tightness in specific organs and tissues [10]. Although forming a continuum throughout the vascular system, endothelial cells have important structural differences depending on the type and size of vessels as well as tissue localization [11,12]. For example, in larger arteries or BBB, the endothelium is continuous and tight. In capillaries and lymphatics, endothelial cells are rather discontinuous. Some specialized vessels have a fenestrated endothelium, for example the sinusoids of the liver [11,12]. 

Conceptually, there are two ways by which molecules can pass the endothelium barrier, namely the paracellular pathway and the transcellular pathway. In the discontinuous endothelium, the paracellular pathway is dominant and allows passive filtration of macromolecules according to size and concentration [13]. Paracellular passage also occurs in continuous endothelium by the opening of junctions in response to signaling events; however, paracellular transport through the BBB is considered as inexistent under physiological conditions [14]. Alternatively, macromolecules such as HDL can surmount the endothelial barrier by transcellular pathways. Several modalities are defined: (i) passive diffusion of lipophilic molecules through the membrane bilayer [15]; (ii) protein carrier mediated transport for solutes and nutrients such as glucose or fatty acids [16]; (iii) formation of channels through the cytoplasm whose lids need to be opened first [17]; and (iv) receptor mediated endocytosis for subsequent vesicular transport and exocytosis on the contralateral side [6]. In addition to limiting local exchanges of molecules and cells between tissue and blood, the endothelium thereby also regulates many systemic processes, including volemia and blood pressure, hemostasis, and host defense [18]. 

**Figure 2:**
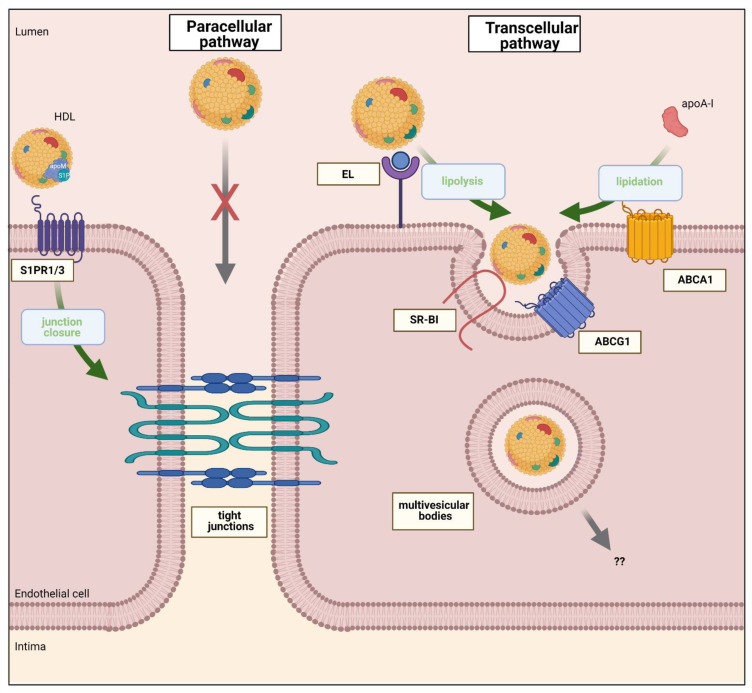
Transendothelial transport of HDL. S1P bound to HDL induces endothelial cells junction closure preventing paracellular pathways. HDL binds to EL that lipolyses it to generate HDL particles with smaller stoke diameter. Resulting HDL binds to SR-BI and ABCG1 and is internalized. Lipid-free apoA-I is lipidated by ABCA1 before being transported like HDL. Internalized HDL is trafficked via endosome and multivesicular bodies before being exocytosed via an unknown pathway.

Early studies in animals and humans measuring the clearance of plasma proteins and lipoproteins from lumen into the arterial wall or into the lymph found that transport rates were positively and inversely correlated with the concentration and diameter of these macromolecules, respectively [19,20]. It was therefore suggested that the transendothelial transport of plasma proteins including low-density lipoprotein (LDL) and HDL happened by ultrafiltration through inter- or intracellular pores of the endothelium. However, more recent studies provided several lines of evidence suggesting that the transendothelial transport of HDL is actively regulated rather than passive. When cultured in a transwell system, human (HAEC) and bovine (BAEC) aortic endothelial cells express intercellular junctions and form an impermeable barrier like in vivo [21,22,23]. In these conditions, HDL added to the apical compartment is found in the lower compartment after 30 min, a process inhibited when lowering the temperature to block cellular activity [21]. Importantly, when using fluorescent and electron microscopy, tagged-HDL is only found within intracellular vesicles and not in between endothelial cell junctions, further supporting the model of a transcellular transport of HDL [22,24,25]. In both HAECs and human brain microvascular endothelial cells (HBMEC), HDL is recovered in endosomes but colocalizes with neither caveolin 1 nor clathrin. [22,25]. However, in human umbilical vein endothelial cells (HUVEC), HDL colocalizes with clathrin [24]. Binding, uptake and transport are competed by an excess of unlabeled HDL, demonstrating their specificity in endothelial cells [21]. In vitro experiments using specific small interfering (si)RNA or pharmacological inhibition showed that HDL binding to, internalization into, and transport through BAEC and HAEC involve at least three proteins; (i) scavenger receptor (SR)-BI (encodes by *SCARB1*), (ii) endothelial lipase (EL, encodes by *LIPG*), and (iii) ATP binding cassette (ABC)G1 (Figure 2) [21,26]. 

Fung and colleagues confirmed the limiting role of SR-BI in HDL transcytosis through HBMEC [25]. Surprisingly, they found that HDL uptake by HBMEC is not limited by the adaptor protein Na(+)/H(+) exchange regulatory cofactor NHE-RF3 (PDZK1), which was shown to limit SR-BI cell surface expression in several cells. In lymph endothelial cells, Lim et al. reported that fluorescent HDL or radioactive cholesterol injected into the skin of *scarb1* knockout mice is removed and recovered more slowly in the lymph nodes, thoracic duct, and blood as compared to C57/BL6 WT controls [27]. These results were further confirmed using a specific SR-BI blocking antibody and importantly the effect was similar to surgical interruption of lymphatic drainage. These data suggest that SR-BI also limits the uptake of HDL from tissue into lymph vessels. Altogether, SR-BI appears to be an important regulator of transendothelial HDL transport. SR-BI also facilitates the binding and transendothelial transport of LDL, both in vitro and in vivo, via the adapter protein dedicator of cytokinesis (DOCK)4, which binds to the cytoplasmic carboxyterminal domain of SR-BI [28,29]. Importantly, HDL transendothelial transport is independent of DOCK4 [28]. Finally, it is important to note an alternative model by which endothelial SR-BI was suggested to enhance RCT by facilitating cholesterol transfer from the subendothelial space to HDL without entering the arterial wall [30].

The transendothelial transport of HDL through BAEC is reduced and enhanced by the knockdown and overexpression of *LIPG*, respectively [26]. The joint knockdown of both *SCARB1* and *LIPG* did not reduce the transport more profoundly than the interference with each gene individually [26] suggesting that SR-BI and EL act in series or cooperatively rather than in parallel on two independent pathways. In contradiction of such interaction, EL-modified HDL exhibits diminished SR-BI-dependent cholesterol efflux from cholesterol loaded COS-7 cells [31]. Interestingly, the transfection of BAEC with a catalytically inactive EL enhanced HDL binding but neither uptake nor transport [26]. These data suggest that EL promotes transport indirectly by mediating HDL binding and remodeling or by releasing signaling molecules. In line with this interpretation, siRNA against *LIPG* or inhibition of EL’s lipolytic activity prevented the decrease of HDL size during transendothelial transport. Interestingly, the knockdown of *SCARB1* did not prevent the reduction of HDL’s stoke diameter [26]. However, HDL2 and HDL3 are transported with similar efficiency, confirming the view that solely reducing HDL size does not improve transendothelial transport. 

Several factors upstream of receptors or transporters that are known to interact directly with HDL were found to promote transendothelial HDL transport. ABCA1 limits transendothelial transport of lipid-free apoA-I by promoting lipid efflux and the formation of nascent lipidated HDL particles to interact with ABCG1 and SR-BI [32]. ApoA-I also activates the ectopic beta-ATPase on BAEC surface to form ADP, which in turn, by signaling through the purinergic receptor P2Y1, activates endocytosis of HDL by an as yet unknown low affinity-binding site [33]. Vascular endothelial growth actor (VEGF) promotes the uptake and transport of HDL through HAECs by inducing the translocation of SR-BI to the cell surface [23]. Finally, interleukin (IL)-6 promotes HDL endothelial uptake and transport by inducing *LIPG* expression [26]. Estradiol inhibited endothelial SR-BI through activation of the G-protein coupled estrogen receptor and thereby LDL transport [34]. Similarly, the proinflammatory mediator high mobility group box 1 (HMGB1) regulated LDL transcytosis via SR-BI [35]. Whether these pathways also influence HDL transport is unknown and remains to be investigated. 

## 3. Promotion of Endothelial Function and Integrity by HDL

HDL exerts many protective effects on the integrity and function of the endothelium. HDL helps to maintain the integrity of the endothelium by promoting junction closure [36], by preventing the loss of glycocalix through proteoglycan shedding [37,38], by inhibiting apoptosis, and by stimulating the proliferation and migration of both mature endothelial cells and endothelial progenitor cells (EPC) [39,40]. HDL inhibits leukocyte diapedesis through the endothelium by suppressing the expression of selectins as well as VCAM1 and ICAM1 [41]. HDL supports endothelial-dependent vasodilation by promoting the phosphorylation of eNOS and thereby NO production [42]. Most of these protective functions have been shown in vitro as well as in animal models. The vasodilating effects of HDL have also been demonstrated in humans both by observational and interventional strategies. Patients with low or even absent HDL due to mutations in *APOA1* or *ABCA1* presented with reduced flow-mediated vasodilatation of the brachial arteries [43,44]. Flow-mediated vasodilation was restored in these patients as well as in patients with coronary heart disease after infusion of artificially reconstituted HDL (rHDL) [45]. 

**Figure 3:**
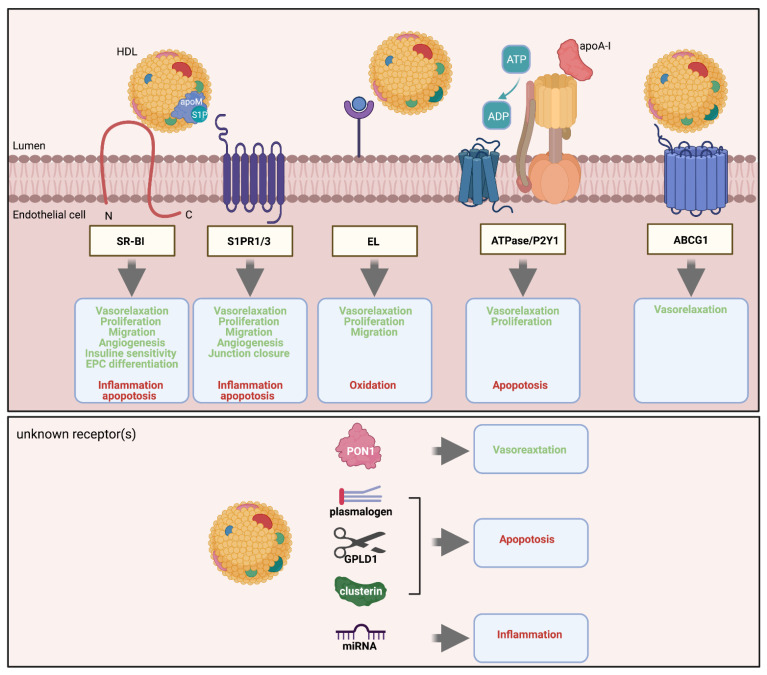
Signaling pathways of HDL in endothelial cells. HDL binding to SR-BI induces several vasoprotective functions (green) and suppresses harmful functions (red). In addition, SR-BI may help to facilitate the presentation of apoM-bound S1P to S1PR1 or S1PR3. EL hydrolysis of HDL-associated lipids supports HDL signaling through S1PR. Efflux of cholesterol to HDL by ABCG1 promotes NO-induced vasorelaxation. Lipid-free apoA-I binds to the ectopic ATPase inducing ATP to ADP conversion and subsequent signaling via P2Y1. Finally, several HDL-associated molecules, including PON1, plasmalogens, GPLD1, and clusterin, promote vasoprotective function via unknown mechanisms.

Several agonists carried by HDL and receptors involved in transendothelial HDL transport are responsible for the protective functions of HDL on the endothelium (Figure 3). Currently, it is not well understood whether this redundancy reflects parallel activities or interactions of different pathways. 

### 3.1. SR-BI Mediated HDL Signaling in Endothelial Cells

SR-BI is a widely expressed multiligand transmembrane glycoprotein. In endothelial cells it is found on both apical and basolateral plasma membranes and also intracellularly. In addition to the canonical ligand HDL, SR-BI binds dozens of macromolecules including native and modified LDL as well as viruses such as hepatitis C and SARS-CoV-2 [46,47]. As the canonical function, SR-BI mediates selective lipid uptake from HDL into cells as well as cholesterol efflux from cells to HDL [47], probably depending on the concentration gradient of cholesterol. Especially in endothelial cells, SR-BI exerts additional functions. In addition to transendothelial transport, HDL binding to SR-BI triggers several intracellular signaling events. The adapter protein PDZK1 stabilizes SR-BI expression at the plasma membrane by interaction with the three-carboxyterminal amino acid residues of SR-BI [48]. PDZK1 further helps the recruitment and phosphorylation of the non-receptor kinase src and subsequent activation of the serine/threonine-protein kinase B (Akt) and mitogen-activated protein kinase (MAPK) to downstream activation of eNOS generating NO and vascular relaxation [49]. This process is partially suppressed upon siRNA knockdown of *SCARBI* or *PDZK1* [50]. Similarly, the induction of SR-BI by statins enhances eNOS activation [51]. 

MAPK activation also activates the small G-protein Rac1 promoting lamellipodia inducing endothelial cell migration and re-endothelialization [52]. Another signaling pathway includes the recruitment and activation of the serine/threonine liver kinase B1 (LKB1) by PDZK1 that phosphorylates AMP-activated protein kinase (AMPK) for downstream phosphorylation of phosphatidylinositol 3-kinase (PI3K) and subsequent activation of Akt [48]. The signaling cascade then activates: (i) cyclooxygenase-2 (COX-2) producing prostacyclin (PGI_2_) to increase vascular compliance [53], (ii) glucose transporter (GLUT)4 mediating cellular glucose uptake and enhancing insulin sensitivity [54], as well as (iii) ubiquitin ligases Siah 1 and 2 stabilizing hypoxia-inducible factor-1α (HIF-1α), a transcription factor regulating angiogenesis [55]. 

### 3.2. S1P Receptors Mediated HDL Signaling in Endothelial Cells

The sphingosine-1-phophate receptors (S1PR) are G-protein-coupled receptors. Three of them, S1PR1, S1PR2, and S1PR3, are expressed by endothelial cells. S1PRs regulate a wide range of cellular and cytoprotective functions including cell motility, cell proliferation and survival, cytoskeletal rearrangement and endothelial barrier integrity [56]. Sphingosine-1-phosphate (S1P) is the natural ligand of S1PRs and 50%70% of S1P in plasma is transported by HDL, predominantly by the minor subfraction that contains apoM [57,58]. The binding of S1P by this lipocalin not only leads to its enrichment in HDL but also facilitates the activation of S1P receptors. ApoM-bound and therefore HDL-carried S1P, but not albumin-bound S1P, inhibits monocyte binding to endothelial cells by suppressing VCAM1 and ICAM-1 expression [59,60]. The importance of apoM for S1P transport and for maintaining endothelial barrier function, at least in the microvasculature, is highlighted in *apom* knockout mice that present with severely reduced plasma levels of S1P and exudation of plasma into extravascular tissues [58,61]. 

HDL promotes endothelial barrier integrity of HUVEC by a process involving S1PR1 and Akt activation [36]. Interestingly these effects last longer when S1P is associated with HDL rather than with albumin [62]. ApoA-IV was also identified as a chaperon for S1P in *apom* and *alb* double knockout mice. When bound to recombinant apoA-IV, S1P induces endothelial barrier formation but only to the level of S1P bound to albumin [63]. Recently, Dennhardt and colleagues showed that the promotion of barrier integrity in human microvascular endothelial cells by S1P signaling involves AMPK phosphorylation [64]. The role of S1PR1 in barrier integrity was further put into perspective as endothelial specific *s1pr1* knockout mice show BBB increased permeability to small (<3 kDa) but not to larger molecular weight (<10 kDa) tracers [65]. Similarly, BBB leakages toward sodium fluorescein (365 Da) and Alexa fluor488 (643 Da) but not FITC-dextran (10 kDa) are increased in *apom* knockout mice. These leakages are rescued by treatment with the S1PR agonist SEW2871 [66].

The anti-apoptotic properties of HDL also depend on apoM-S1P complex and S1PR1 and S1PR3 signaling [67]. In fact, inhibition of apoptosis, either induced by oxidized LDL or by starvation, were the first examples illustrating that HDL exerts biological activities via lysosphingolipids including S1P [68,69]. HDL-S1PR signaling promotes Akt and extracellular signal-regulated kinase (ERK) phosphorylation and further phosphorylates the pro-apoptotic protein BAD promoting its dissociation from B-cell lymphoma-extra-large (BCL-X_L)_ and subsequently inhibiting mitochondrial pathway of apoptosis [68,69]. This process is mimicked by lysosphingolipids; sphingosylphosphorylcholine and lysosulfatide present on HDL [69]. 

HDL signaling through S1PR1 and S1PR3 also reduces nuclear factor-kappa B (NF-κB) activation in HAEC, HUVECs and HBMEC, and thereby decreases cell surface expression of VCAM1, ICAM1, and E-selectin as well as subsequent monocyte adhesion [50,59,60,70]. Similarly, cord-blood HDL signaling via S1PR1 reduces NF-κB activation and subsequent expression of VCAM1, ICAM1 and E-selectin as well as secretion of IL-8 and monocyte chemoattractant protein 1 (MCP1) by placental endothelial cells [71].

The activation of S1PR3 by HDL-associated S1P promotes endothelium-dependent vasodilation via phosphorylation of Akt and eNOS [72]. Similarly, induction of S1PR1 by statins enhances HDL-induced eNOS activation [73]. Fenofibrate treatment of *ldlr* knockout mice increases plasma levels of both HDL cholesterol and S1P as well as the expression of S1PR1and S1PR3 and the activity of Akt and eNOS in the aorta. In parallel with these effects, fenofibrate prevents the development of abdominal aortic aneurysm induced by infusion of angiotensin II [74]. 

Finally, HDL promotes angiogenesis of HUVEC through increasing the expression and phosphorylation of VEGF receptor 2 (VEGFR2) by a mechanism that involves the activation of S1PR3 but neither S1PR1 nor S1PR2 [75]. Interestingly, the tumor vessels of endothelial specific *s1pr1* knockout mice have decreased endothelial barrier function and increased vascular sprouting and branching, while the overexpression of S1PR1 leads to the opposite phenotype [76]. Knocking out *s1pr2* or/and *s1pr3* further exacerbates the vascular abnormalities, further supporting the role of S1PRs in angiogenesis [76]. 

Interference with the presence of S1P or the activity of S1PR1 or S1PR3 mimics many effects seen by interference with SR-BI. This raises the question whether SR-BI- and S1PRs-mediated signaling of HDL act in parallel or interact. In support of interaction, it has been suggested that SR-BI tethers HDL on the cell surface and thereby increases the likelihood of S1P/S1PR interaction [77,78]. Moreover, our lab showed that siRNA mediated knockdowns of S1PR1 or S1PR3 reduce the expression of SR-BI [79]. 

### 3.3. ABCG1 Mediated HDL Signaling in Endothelial Cells

ABCG1 is composed of six transmembrane domains and requires the formation of dimer to be functional. In addition to facilitating transendothelial transport of HDL, ABCG1 contributes to HDL-induced NO production. By promoting cholesterol efflux to HDL and thereby decreasing cholesterol levels in plasma membrane rafts, ABCG1 releases the inhibitory interaction of eNOS with caveolin-1 in endothelial cells and hence induces eNOS phosphorylation [80,81]. Whether ABCG1 has other direct or indirect signaling roles remains to be investigated. 

### 3.4. EL Mediated HDL Signaling in Endothelial Cells

EL is a member of the triglyceride lipase family with phospholipase activity [82]. EL is primarily expressed and secreted by endothelial cells. It is either bound to heparan sulfate proteoglycans (HSPG) covering the luminal cell membrane of endothelial cells or circulated in the plasma [83]. EL binds HDL with high affinity and hydrolyzes its phospholipids to generate free-fatty acids and lysophospholipids. As a result, the stoke diameter of HDL decreases [26]. Several studies showed that remodeling by EL improves several vasoprotective functions of HDL. EL increases the anti-oxidative capacity of HDL, probably independently of paraoxonase 1 (PON1), whose abundance is decreased and increased in HDL of rodents and humans, respectively [84,85]. HDL-induced angiogenesis is markedly decreased in *lipg* knockout mice compared to wild-type control as well as in BAEC after siRNA interference against *LIPG* [86]. The authors reported that EL-modified HDL also increases eNOS and Akt phosphorylation as well as subsequent endothelial migration and angiogenesis by increasing the signaling of HDL-bound S1P via S1PR1 but not S1PR3. Similarly, Radulovic and colleagues showed that EL-modified HDL promotes the enrichment of the plasma membrane with eNOS and subsequent eNOS phosphorylation in EA.hy 926 cells, as well as aortic relaxation in c57BL/6 mice [84]. Furthermore, compared to untreated control HDL, EL-modified HDL reduces the content of free cholesterol in the plasma membrane and increases the cellular concentration of cholesteryl ester. Finally, the lysophospholipids and free-fatty acid generated by EL lipolysis may exert regulatory effects on endothelial cells. In particular, Riederer et al. reported that EL-generated lysophosphatidylcholines promote IL-8 expression in endothelial cells [87]. Whether this and other lipolytic products are responsible for other vasoprotective functions of HDL remains to be studied. 

### 3.5. ApoA-I/ecto-F1-ATPase/Purinergic Receptor Signalling in Endothelial Cells

Mitochondrial ATP synthase has been recently detected at the surface of different cell types including endothelial cells [33]. In this ectopic localization, however, the enzyme acts as an ATPase and generates ADP that activates the purinerergic receptor P2Y1 [88]. The activation of ectopic beta-ATPase by apoA-I in HUVECs inhibits apoptosis, promotes cell proliferation, and induces eNOS phosphorylation by a mechanism involving P2Y1 and the serial phosphorylation of the class I PI3Kβ and Akt [89,90,91]. Inhibitors of F1-ATPase, such as inhibitory factor 1 (IF1) and oligomycin, as well as P2Y1 antagonists, completely block apoA-I-induced Akt phosphorylation as well as thromboxane-induced vasorelaxation of mouse aortas [89,91]. IF1 and oligomycin also inhibit EPC proliferation, suggesting a role of the apoA-I/ecto-ATPase/P2Y1 axis in angiogenesis [92]. 

### 3.6. Identification of HDL-Associated Agonists by Proteomics, Lipidomics or Transcriptomics

Recent systems biology approaches identified several proteins, lipids, and microRNAs (miRNA) in HDL that affect the function or survival of endothelial cells. Their mode of action is little understood. Bioinformatic analyses of proteomes and endothelial functionality of HDL isolated from several dozens of donors found significant correlations between the anti-apoptotic activity and the content of HDL in clusterin (apoJ) or glyosylphosphatidylinositol phospholipase D1 (GPLD1) [2,93]. The same strategy found PON1 as a determinant of HDL’s activity to activate eNOS [94]. Supporting the anti-apoptotic effect of clusterin, the survival of starving HAECs is dose-dependently enhanced by the enrichment of HDL with increasing amounts of clusterin, and is decreased by parallel incubation with anti-clusterin antibodies. The anti-apoptotic activity of clusterin appears to involve Akt phosphorylation [93]. Likewise, anti-GPLD1 antibodies reduce the ability of HDL to inhibit starvation-induced apoptosis of HAECs [2]. Conversely, artificial rHDL containing GPLD1 in addition to apoA-I and phosphatidylcholine shows a higher anti-apoptotic activity towards HAECs than rHDL containing apoA-I and phosphatidylcholine only [2]. It is important to note that GPLD1 is not able to inhibit apoptosis in the absence of HDL. These findings raise the hypothesis that HAECs express a GPI-anchored protein that regulates their cell death or survival. The cleavage of the GPI-anchor by GPLD1 either activates a survival pathway or interrupts a cell death pathway. The intracellular signaling by which GPLD1 modulates the anti-apoptotic actions of HDL remains to be further investigated.

The combination of lipidomic and functional analyses of HDL also found correlations between the anti-apoptotic activity and the content of HDL in phosphatidylcholine plasmalogens as well as sphingomyelins SM42:2 and SM42:3 [2,95]. Supporting the importance of the enrichment of rHDL with either plasmalogen PC35:1 or SM42:2 or SM42:3 increases the anti-apoptotic activity of rHDL. The mechanisms by which plasmalogens and sphingomyelins enhance the anti-apoptotic activity of HDL are unknown. 

In addition to numerous proteins and lipid species, HDL particles carry miRNAs, small non-coding RNA that have emerged as cellular regulators. Several miRNAs have reported functions on angiogenesis, vascular inflammation and atherosclerosis, vascular tone and endothelial barrier [96]. Interestingly the level of miRNA in HDL correlates with vascular disease risk [97]. One of the most abundant miRNAs in HDL is miR-233, which was recently shown to affect gene expression in human coronary artery endothelial cells. For example, ICAM-1 expression is suppressed by miR-233 [98]. It is currently not known how miRNAs are transferred from HDL into endothelial cells. It has also been generally questioned whether the low copy numbers of miRNAs physiologically carried by HDL and transferred to endothelial cells are sufficient for the posttranscriptional regulation of endothelial function [99].

## 4. HDL and Endothelial Cell Dysfunction

More than 25 years ago, Fogelman’s lab created the concept of HDL dysfunction when they showed that HDL isolated from patients as well as experimental rabbits with acute phase response lost their activity to suppress MCP1 expression by endothelial cells [100]. In their pioneering experiments, Fogelman and colleagues already identified the enrichment of HDL with serum amyloid A (SAA) and loss of apoA-I and PON1 as important contributors to HDL dysfunction. Thereafter many laboratories reported that various inflammatory or cardio-metabolic diseases such as coronary artery disease (CAD), type 2 diabetes mellitus (T2DM), obesity, and chronic kidney disease (CKD) impair HDL’s multifaceted endothelial-protective functions and ultimately render HDL dysfunctional [101,102]. Table 1 summarizes the most important observations divided by disease. Overall, it is important to emphasize that HDL dysfunction can result from deprivation or molecular modification of protective agonists, as described in Section 2, or the enrichment of HDL with adversely acting components [103].

### 4.1. Coronary Artery Disease

Besler et al. showed that HDL isolated from patients with CAD (CAD-HDL) fails to stimulate Akt-dependent phosphorylation of eNOS^ser1177^ but instead enhances inhibitory phosphorylation of eNOS^Thr495^ [94]. Mechanistically, CAD-HDL is enriched with the oxidation marker malondialdehyde due to reduced anti-oxidant activity of HDL-associated PON1. CAD-HDL activates the lectin-like oxidized LDL receptor 1 (LOX-1) triggering protein kinase (PK)CβII activation and subsequent phosphorylation of eNOS^Thr495^ suppressing NO production. Subsequently, the NO-dependent anti-inflammatory and repair–stimulating effects of HDL towards the endothelium are compromised [94]. Furthermore, CAD-HDL contains four to five times less S1P than HDL of healthy donors. The loading of CAD-HDL with S1P restores the NO-dependent vasodilator capacity of CAD-HDL on rat mesenteric arteries and restores HDL ability to phosphorylate ERK1/2, Akt and eNOS^ser1177 104^ in human endothelial cells. In parallel with the reduced ability to induce NO production, CAD-HDL has also lost anti-thrombotic properties [105]. Compared to the HDL of healthy donors, CAD-HDL has reduced capacities to inhibit the endothelial expression of pro-thrombotic mediators, such as tissue factor and plasminogen activator inhibitor type 1, and to activate anti-thrombotic factors such as tissue factor pathway inhibitor and tissue plasminogen activator. HDL isolated from patients with acute coronary syndrome promoted laser-injured carotid artery arterial thrombus formation in mice [105].

Myeloperoxidase (MPO) strongly contributes to oxidative modifications of CAD-HDL. MPO catalyzes the nitration and chlorination of apoA-I and thereby impairs its ability to induce ABCA1-dependent cholesterol efflux from macrophages [123]. Under a variety of inflammatory conditions, and particularly in CAD, MPO promotes the oxidative modification of methionine residues in PON1 so that its anti-oxidative activity is compromised [106]. Thus, MPO, PON1, and HDL form a ternary binding complex, wherein MPO and PON1 reciprocally modulate each other’s function.

Moreover, in contrast to the HDL of healthy control subjects favoring anti-apoptotic pathways, CAD-HDL favors pro-apoptotic signaling. This endothelial dysfunction is associated with a reduced content of clusterin and increased content of apoC-III in CAD-HDL [93]. A lack of HDL-associated clusterin leads to inefficient stimulation of PI3K/Akt and subsequently reduced expression of the antiapoptotic Bcl-xL. In parallel, the elevated content of CAD-HDL in apoC-III favors pro-apoptotic signaling in HAECs by phosphorylation of p38-MAPK and up regulation of Bcl-2 protein tBid. This dysfunction, in addition to the reduced cholesterol efflux capacity of apoC-III –enriched HDL [93], may be the reason for the observation that cholesterol in apoC-III-free HDL but not cholesterol in apoC-III-containing HDL is inversely associated with the incidence of major adverse cardiovascular event (MACE) in several cohort studies [124]. It also raises the question of whether inhibition of ApoC-III by antisense-oligonucleotides not only lowers triglycerides and increases HDL-cholesterol levels but also improves HDL-function [125]. Most recently, Tiedge and colleagues reported for the first time on the association of an endothelial HDL dysfunction with incidence of MACE, defined as the combination of death from cardiovascular causes, ischemic heart disease, nonfatal myocardial infarction, and coronary revascularization [107]. In a nested case control study of 340 persons with MACE and 340 matched controls without MACE during more than 10 years of follow up, HDL’s ability to suppress TNF<-induced mRNA expression of VCAM1 in HUVEC was inversely associated with the incidence of MACE. This association was independent of other risk factors including the cholesterol efflux capacity of the apoB-depleted plasma [107].

### 4.2. Diabetes

In addition to the reduced cholesterol efflux capacity, HDL of patients with type 1 or type 2 diabetes are characterized by the impairment of several endothelial functions. Compared to HDL of non-diabetic subjects, HDL of T2DM patients are less capable to promote NO production and to inhibit ROS production as well as the expression of adhesion molecules [109]. Normal endothelial HDL functions are restored by several interventions including healthy life style, bariatric surgery, and treatment with liraglutide or niacin [108,126,127]. The reduced eNOS activation is assigned to the higher content in lipid peroxides [108,109]. The reduced anti-apoptotic activity is associated with the loss of GPLD1 and sphingadienine-based sphingomyelins [2]. 

Glycation of plasma proteins and formation of advanced glycation end-products are typical events in diabetes. Glycation of HDL induces the loss of its ability to counteract the inhibitory effect of oxidized LDL on endothelium-dependent vasorelaxation [128]. Glycated HDL particles induce endothelial cell apoptosis and increase oxidative stress [110]. Glycation of apoA-I interferes with its ability to inhibit the expression of VCAM1 and ICAM1 as well as neutrophil adhesion in rabbits and in cell culture [129]. PON1 glycation induces endothelial endoplasmic reticulum stress [111]. In vitro glycation results in impaired S1P binding to HDL and faster S1P degradation due to polymerization of apoM. Similarly, the apoM level is reduced in T2DM patients compared to healthy sex but not age matched controls, but apoM in T2DM HDL was not polymerized [130]. Reports on the content of S1P in HDL of diabetic subjects are controversial. Both lower and higher contents of S1P in HDL of diabetic subjects than in HDL of healthy control subjects were reported [131,132,133]. Whether the reduced apoM level in T2DM is responsible of reduced HDL functions in diabetes remains to be determined. 

### 4.3. Chronic Kidney Disease 

In CKD, HDL accumulates SAA, pulmonary surfactant protein B (PSPB), lipoprotein associated phospholipase A2, apoC-III and symmetric dimethylarginine (SDMA) [112,134,135]. In CKD patients undergoing hemodialysis, HDL-associated SAA and PSPB are associated with cardiovascular events and all-cause mortality independently of HDL-C plasma concentrations [112,136]. In particular, SAA accumulation blunts HDL’s- protective properties towards endothelial cells, such as the promotion of NO production and the inhibition of ROS formation and expression of pro-inflammatory adhesion molecules [114]. 

The high SDMA content makes CKD-HDL activate Toll-like receptor-2 (TLR-2) that inhibits the phosphorylation of Akt and eNOS, and hence NO production, but promotes activation of JNK and thereby NADPH oxidase-dependent superoxide production [112]. Reduced NO bioavailability along with enhanced superoxide production also impair endothelial repair after injury. SDMA-enriched HDL also fails to suppress the expression of adhesion molecules and monocyte binding to HAEC. These dysfunctions of HDL are present in early CKD stages and become more severe with declining renal function. They are only partially reversed after kidney transplantation. Similar SDMA-dependent HDL function abnormalities were observed in children with CKD but no concomitant disease affecting HDL function [113]. Most recently, the high SDMA content of HDL from CKD patients was reported to promote the breakdown of endothelial glycocalyx, the carbohydrate-rich gel-like protective barrier on the luminal side of the endothelium [38]. Intravital microscopy of the cremaster muscle microcirculation in mice revealed that injection of CKD HDL as well as SDMA promotes the thinning of the glycocalix and subsequently leukocyte rolling. Mechanistic studies in the endothelial cell line EA.hy926 revealed that both CKD-HDL and SDMA damage the endothelial glycocalyx by activation of matrix metalloproteinase-9 (MMP-9) through the induction of TLR-2-signaling. 

Carbamylation is a typical posttranslational protein modification observed in patients with CKD, most prominently in end stage renal disease (ESRD) [137]. In ESRD patients, HDL-associated PON1 activity is decreased and negatively correlated with carbamylation of HDL [115]. HDL of ESRD patients as well as artificially carbamylated HDL inhibits the proliferation and migration of HAECs as well as angiogenesis, possibly by decreasing VEGFR2 and SR-BI signaling pathways [115]. 

### 4.4. Chronic Inflammatory Diseases

Low HDL-cholesterol levels are associated with increased risk of several autoimmune diseases [138]. In addition, HDL lose their protective vasoprotective properties in patients with chronic immune-inflammatory disease such as rheumatoid arthritis (RA), systemic lupus erythematosus (LE) and psoriasis [139]. In women with LE and antiphospholipid antibodies, carotid intima media thickness and pulse wave velocity are inversely correlated with HDL-PON1 activity [116]. Moreover, unlike HDL of healthy donors, HDL of LE patients inhibits endothelial NO production and promotes VCAM-1 expression, superoxide production, and monocyte adhesion by HAEC. In RA, adding the anti-inflammatory TNFα-inhibitor infliximab to the standard treatment with methotrexate improves but does not completely restore the ability of HDL to promote NO production and to inhibit superoxide production and endothelial apoptosis [117]. In psoriasis patients, the higher MDA content blunts HDL-mediated inhibition of TNFα-induced monocyte adherence to endothelial cells and endothelial apoptosis [118]. Moreover, HDL-dependent RCT is inversely associated with Disease Activity Index for Psoriatic Arthritis in psoriasis patients, strengthening the role of inflammatory activity as a key factor in the development of HDL dysfunction and promotion of accelerated atherosclerosis in these patients [140]. 

Even a low chronic systemic inflammation such as the one associated with periodontitis impairs HDL-dependent RCT, endothelial NO synthesis, and PON1 activity while promoting endothelial superoxide oxide production [119]. Probably due to the acute inflammatory stimulus, the aforementioned HDL properties are transiently further impaired by periodontal treatment but restored to baseline levels six months post dental care. The worsening of HDL function upon periodontal treatment is associated with remodeling of the HDL proteome with increased levels of SAA and complement factor C3. Interestingly, there was no difference in HDL-RCT in patients with periodontitis at any timepoint in this study, suggesting the heterogenous impact of inflammation on the different vascular effects of HDL. In agreement with this, our lab previously found different determinants of HDL functions and excluded cholesterol efflux capacity as a proxy of other HDL-functionalities [2]. 

### 4.5. Infections and Sepsis

Both low and high levels of HDL cholesterol are associated with increased risk of infectious diseases of either bacterial or viral origin [141]. Clinical data showed that HDL-C levels decrease rapidly during sepsis and that low HDL-C levels are associated with increased morbidity and mortality [142,143]. The results of Mendelian randomization studies indicate genetic causality for the associations of HDL-C with both incident infections and prognosis in sepsis [144]. 

Together with HDL-C, the plasma levels of S1P as well as PON1 are also significantly reduced in sepsis patients [145,146]. S1P concentrations of HDL are inversely correlated with endothelial damage and dysfunction both in septic patients and in a rat model of sepsis [120]. HDL-S1P injection significantly reduces pulmonary edema and endothelial leakage in septic rats. In vitro, HDL-S1P promotes the proliferation and migration of pulmonary endothelial cells [120]. HDL from septic patients with acute respiratory distress syndrome are characterized by the loss of apoA-I and PON1 as well as the enrichment with SAA, apoC-III and apoE. Interestingly, the injection of HDL from septic ARDS patients exacerbates the pulmonary endothelial dysfunction and acute lung injury induced by cecal ligation and puncture (CLP) in wild type and *apoa1* knockout mice [121] 

### 4.6. HDL, Endothelial Cells and COVID-19

The COVID-19 pandemic has caused more than 3.1 million deaths worldwide (27 April 2021, https://covid19.who.int/). Although primarily targeting the respiratory tract, SARS-CoV-2 also affects the heart and the vasculature [147]. Endothelial cells are infected directly by SARS-CoV-2 due to the abundant endothelial expression of the membrane bound receptor angiotensin-converting enzyme 2 (ACE2) [148,149]. Once dysfunctional, endothelial cells exert pro-inflammatory, pro-oxidant and pro-thrombotic actions, thus contributing to the generalized inflammatory cytokine storm and perpetuating a vicious cycle [150]. Recent evidence has suggested that the spike (S) protein of SARS-CoV-2 has specific affinities for cholesterol and possibly HDL components. Furthermore, SR-BI significantly facilitates entry of the virus into liver Huh-7 cells, acting as an important cofactor of ACE2 by stabilizing the attachment of the viral particles to the cell membrane [46]. The pharmacological inhibition of SR-BI activity significantly reduces the infection of this hepatocyte cell line with SARS-CoV-2 [46]. Considering the high expression of SR-BI on endothelial cells [151], HDL may interfere with the endotheliitis induced by SARS-CoV-2 infection.

Whereas plasma levels of LDL-C and triglycerides already drop during early phases and in mild courses of COVID-19, HDL-C levels are significantly decreased only in severe cases [152]. Dysfunctional HDL were reported in severely ill COVID-19 patients along with major changes in the HDL proteome. A decreased content in PON1 and an increased content of SAA and alpha-1 antitrypsin compared to control subjects are associated with impaired ability of HDL-mediated to inhibit the apoptosis of endothelial cells [122]. In vitro, glycation impairs the inhibitory activity of HDL towards SARS-CoV-2 infection of epithelial cells [153]. Whether or not infection of endothelial cells with SARS-CoV2 is inhibited by HDL and disturbed by glycation remains to be investigated. 

## 5. HDL and Blood–Brain Barrier Function in Stroke and Neurodegenerative Diseases

Several but not all studies found that HDL-C is inversely associated with the risk of stroke [154,155,156]. HDL perfusion reduces brain edema and promotes BBB integrity [157,158]. In two rat models, the injection of rHDL two hours prior to the induction of stroke reduces the size of the necrotic brain injury, possibly by reducing the release of ROS from endothelial cells [157]. In a subsequent study, the intravenous injection of HDL immediately or up to five hours after stroke reduces brain edema by maintaining BBB integrity and reducing ICAM1 level [159]. In particular, HDL maintains BBB integrity by decreasing the oxygen-glucose deprivation and disorganization of vascular endothelial cadherin [158]. Similarly, the subcutaneous injection of the ApoA-I mimetic peptide L-4F decreases BBB leakage and macrophage infiltration in diabetic db/db mice with stroke [160]. By investigating stroke in endothelium-specific *scarb1* knockout mice, Tran-Dinh and colleagues recently showed that SR-BI contributes to the protection of the BBB by HDL [161]. Subjecting the well described BBB cell line hCMEC/D3 to oxygen-glucose deprivation, they showed that HDL maintains BBB integrity and that blocking selective lipid uptake by BLT1 blocked the protective effects of HDL [161]. Interestingly, HDL isolated up to 4.5 h after stroke are larger and contain less apoA-I and PON1 but more α1antitrypsin (AAT) and MPO. Accordingly, HDL from stroke patients is less effective in reducing TNFα-induced VCAM1 level in endothelial cells [162]. Decreased content in PON1, increased content of AAT, and reduced anti-inflammatory functions of HDL are preserved three months after stroke [163]. 

Plasma levels of HDL-C and apoA-I are also inversely associated with Alzheimer’s (AD) and Parkinson’s (PD) diseases [164,165,166,167]. Several cross-sectional studies showed that PON-1 level and function are reduced in AD patients compared to non-cognitive declined controls [168,169,170,171] whereas a high level of plasma clusterin is associated with increased AD risk [172,173]. Furthermore, *apoa1* knockout mice show increased beta-amyloid (Aβ) deposition within the cerebrovascular wall and increased vascular inflammation. However, other studies reported either no difference [174] or reduced Aβ deposition within the vascular wall [175]. Mice overexpressing human *APOA1* have reduced vascular inflammation and Aβ deposition [176]. Likewise, tail vein injection of rHDL as well as intravenous injections of either clusterin or apoA-I Milano reduce Aβ levels in the brain or vasculature [177,178]. ApoA-I Milano also reduces Aβ induced apoptosis. These data support a role of HDL preferentially at the vasculature. In a transwell system, HDL promotes Aβ transport through hCMEC/D3 in a apoA-I lipidation-dependent manner, with spherical HDL being the most effective [179]. Using a three dimensional (3D) in vitro model of cerebral arteries in AD, Robert et al. showed that HDL induces NO secretion and prevents Aβ-induced inflammation,—the latter independently of eNOS signaling [180]. This process involves SR-BI, which blocks Aβ uptake into BBB endothelial cells. However, the inhibition of SR-BI mediated selective lipid uptake by BLT1 does not prevent the ability of HDL to reduce Aβ-induced monocyte binding to the endothelial cells. Furthermore, HDL particles enriched in apoE reduce Aβ deposition in the cerebrovascular wall independently of SR-BI while requiring HDL crossing the endothelial barrier [181]. These results are in line with recent epidemiological data showing that HDL containing apoE but not apoCIII is inversely associated with AD risk [182]. Together these results open new questions whether HDL subpopulations differ by their interactions with endothelial cells of the BBB.

## 6. Conclusions

Mediation of efflux and reverse transport of cholesterol from macrophage foam cells to the liver is the most intensively investigated function of HDL. The long duration of research and the large body of data led to the paradigm that these classical functions in cholesterol transport explain the associations of HDL with the risk of atherosclerotic cardiovascular diseases (ASCVD). Accordingly, diagnostic tests such as cholesterol efflux capacity have been developed as proxies of HDL’s anti-atherogenic properties [183,184]. However, HDL exerts many other functions that may explain the inverse associations of HDL-C with ASCVD and several other diseases. Among them, the endothelial interactions of HDL deserve special attention. 

First, the intact endothelium is a prerequisite for the physiological function of all organs. In this regard, it is remarkable that HDL helps to preserve many functions of the endothelium by multiple modes of action involving different molecules carried by HDL as well as several cellular counterparts. This redundancy may indicate the critical importance of HDL for a functional endothelium. Nevertheless, the endothelial functions of HDL appear to be very vulnerable. In diseased individuals, a plethora of modifications in the molecular composition of HDL as well as of HDL components cause loss of protective functions or even gain of adverse functions towards endothelial cells. A close understanding of structure–function relationships underlying the multiple effects of HDL on the endothelium in health and disease may lead to the identification of molecular targets for improved diagnostics or therapy.

Second, even within the classical paradigm of reverse cholesterol transport, it is crucial to better understand the endothelial interactions of HDL. The endothelium forms a barrier between the bloodstream, which contains the majority of HDL, and the macrophages in the atherosclerotic plaque. To mediate cholesterol efflux from macrophage foam cells and to return cholesterol to the liver, HDL has to pass two endothelial barriers to enter and leave the arterial wall. The mechanisms underlying this transendothelial HDL transport have been very little investigated. However, even the currently limited data indicate that this transport is a regulated process rather than passive filtration. Its understanding will be important to improve the diagnostics and treatment of ASCVD and other diseases. Among the latter, diseases of the central nervous system are of special interest because of the tightness of the BBB. As HDL-like nanoparticles can be artificially reconstituted, a better understanding of transendothelial HDL transport also opens avenues towards improved delivery of tracers and drugs into various tissues for diagnostics and therapy, respectively [185,186,187,188]. 

## Figures and Tables

**Table 1:** Overview of investigations on HDL and endothelial cell dysfunction.

Disease Studied.	Experimental/Clinical Investigation	HDL-Endothelial Property in Focus	HDL Main Alteration	Cellular Mechanisms	Ref.
Coronary Artery Disease	clinical, in vitro ECs, rabbit	Anti-inflammatory	HDL in acute phase reaction: ↑ SAA and ceruloplasmin on HDL, ↓ HDL PON-1 activity, ↓ apo A-I levels	ECs: ↑ expression of monocyte chemotactic protein 1	[100]
	clinical, in vitro ECs	clinical, in vitro Ecs	CAD patients: ↑ malondialdehyde (MDA), ↓ HDL PON-1 activity	CAD-HDL activates LOX-1-> ↑ PK CβII activation and ↓ endothelial NO production	[94]
	clinical, in vitro ECs	Nitric oxide (NO) production	CAD patients: ↓ HDL-S1P	CAD-HDL loaded with S1P-> ↑ phosphorylation of ERK1/2, Akt and eNOSser1177 ↑ vasodilation of mesenteric arteries	[104]
	clinical, in vitro ECs, mice	Anti-thrombotic, NO production	CAD patients	↑ tissue factor and plasminogen activator inhibitor type 1, and ↓ tissue factor pathway inhibitor and tissue plasminogen activator. ↑ laser-injured carotid artery arterial thrombus formation in mice	[105]
	clinical, in vitro ECs	Anti-oxidant	CAD patients: ↑ myeloperoxidase (MPO), ↓ HDL PON-1 activity	↑ chlorotyrosine content, site-specific PON1 methionine oxidation, and↓PON1 activity	[106]
	clinical, in vitro ECs	Anti-apoptotic	HDL ↓ clusterin, ↑ apolipoprotein C-III	Lack of HDL- clusterin-> ↓ PI3K/Akt and ↓ endothelial Bcl-xL. High HDL-apoC-III -> ↑ phosphorylation of p38-MAPK and ↑ Bcl-2 protein tBid.	[93]
	clinical, in vitro ECs	Anti-inflammatory	CAD patients	CAD-HDL:↓ inhibition of TNFα-induced endothelial VCAM-1 mRNA expression	[107]
Diabetes mellitus type 2/Obesity	clinical, rats, in vitro ECs	NO production	Obese and post bariatric patients	Glucagon-like-1 treatment in obese rats->HDL-induced endothelial NO production, ↑ vasodilation of aortas	[108]
	clinical, mice, in vitro ECs	NO production, anti-oxidant	T2DM patients:↑MDA and MPO	niacin-treated patients: ↑ vasodilation of mice aortas, ↑ endothelial Repair Capacity of Early EPCs. ↓ Lipid peroxidation->masking positively charged lysine residues	[109]
	clinical, in vitro ECs	Anti-apoptotic, anti-oxidant	T2DM patients: ↓ apoD, ↓ apoM. HDL-S1P controversial	↓ Anti-apoptotic->↓HDL-GPLD1 and sphingadienine-based sphingomyelins. HAECs with glycated-ox-HDL-> ↑ H2O2 and ↓ catalase and Cu(2+), Zn(2+)-superoxide dismutase. Glycation of PON1 induces endothelial ER stress	[2,110,111]
Chronic kidney disease	clinical, in vitro ECs	NO production, anti-oxidant, anti-inflammatory	CKD patients: ↑ symmetric dimethylarginine (SDMA)	CKD-HDL impairs Akt-eNOS-NO production via Toll-like receptor-2 (TLR-2) and induces JNK-NADPH oxidase-dependent ↑ superoxide production	[112,113]
	clinical, in vitro ECs	NO production, anti-oxidant, anti-inflammatory	CAD and CKD patients: ↑ SAA	↓ endothelial NO production, ↓ inhibition of TNFα-induced endothelial VCAM-1 expression, ↑ superoxide production	[114]
	clinical, mice, in vitro ECs	endothelial glycocalyx protection	hemodialysis CKD patients: ↑ SDMA	↓endothelial glycocalyx by activation of matrix metalloproteinases-9 upon induction of TLR-2-signaling	[38]
	clinical, in vitro ECs	Endothelial regeneration, anti-apoptotic, anti-oxidant	End-stage CKD patients: ↑ Carbamylation, ↓ HDL PON-1 activity	↓ proliferation and migration of HAECs->↓VEGFR2 and SR-BI signaling pathways	[115]
Chronic inflammatory disease	clinical, in vitro ECs	Nitric oxide production, anti-oxidant, anti-inflammatory	systemic lupus erythematosus and rheumatoid arthritis HDL: ↓ HDL PON-1 activity	↓ endothelial NO production, blunted TNFα-induced endothelial VCAM-1 expression, ↑ superoxide production	[116,117]
	clinical, in vitro ECs	Anti-inflammatory, anti-apoptotic, anti-oxidant	Psoriasis HDL: ↑MDA, ↓ HDL PON-1 activity	↓ inhibition of TNFα-induced monocyte adherence to ECs and endothelial apoptosis	[118]
	clinical, in vitro ECs	Nitric oxide production, anti-oxidant, anti-inflammatory	periodontitis patients HDL: ↓ HDL PON-1 activity, ↑ SAA and ↑complement factor C3.	↓ endothelial NO production, blunted TNFα-induced endothelial VCAM-1 expression, ↑ superoxide production	[119]
Infection sepsis COVID-19	clinical, rodents models, in vitro Ecs	Anti-inflammatory, anti-apoptotic, anti-oxidant	septic patients: ↓ HDL PON-1 activity and apoA-I levels, ↓ HDL-S1P, ↑ SAA, apoC-III and apoE.	↑ endothelial leakage, ↑ expressions of adhesion proteins and pro-inflammatory cytokines via ↑ NF-κB signaling and ↓ junction protein expression, ↓ proliferation and migration abilities of endothelial cells	[120,121]
	clinical, in vitro ECs	Anti-apoptotic	severely ill COVID-19 patients: ↑ SAA and alpha-1 antitrypsin, ↓ HDL PON-1 activity, ↓ apoA-I levels	↓ inhibition of TNFα-induced endothelial permeability, VE-cadherin disorganization and apoptosis	[122]

Additional abbreviations: EC: endothelial cell, TNFα: tumor necrosis factor alfa, GPLD1: glycosylphosphatidyl-inositol specific phospholipase D1, LOX-1: lectin-like oxidized LDL receptor 1, VEGFR2: vascular endothelial growth factor receptor 2, PKC BII: protein kinase CβII, H2O2: hydrogen peroxide.

## Abbreviations

When the gene names have a different abbreviation, it is given in brackets.

Aβ	beta-amyloid
AAT	α1antitrypsin
ABCA1	ATP binding cassette A1
ABCG1	ATP binding cassette G1
ACE2	angiotensin-converting enzyme 2
AD	Alzheimer’s disease
Akt	protein kinase B
AMPK	AMP-activated protein kinase
Apo	apolipoprotein (M, E or A-IV)
apoA-I (*APOA1)*	apolipoprotein A-I
apoJ	clusterin
ASCVD	atherosclerosis cardiovascular diseases
BAEC	bovine aortic endothelial cells
BBB	blood–brain barrier
BCL-X_L_	B-cell lymphoma-extra large
BLT1	Block-lipid transport 1
CAD	coronary artery disease
CLP	cecal ligation and puncture
CKD	chronic kidney disease
CSF	cerebrospinal fluid
COX-2	cyclooxygenase-2
DOCK4	dedicator of cytokinesis protein 4
EL (LIPG)	endothelial lipase
eNOS	endothelial nitric oxide synthase
EPC	endothelial progenitor cell
ERK	extracellular signal-regulated kinase
ESRD	end stage renal disease
GLUT4	glucose transporter 4
GPDL1	glyosylphosphatidylinositol phospholipase D1
HAEC	human aortic endothelial cells
HBMEC	human brain microvascular endothelial cells
HDL	high-density lipoprotein
HSPG	heparan sulfate proteoglycans
HIF-1〈	hypoxia-inducible factor-1α
HMGB1	high mobility group box 1
HUVEC	human umbilical endothelial cells
ICAM1	induced cellular adhesion molecule 1
IF1	inhibitory factor 1
IL	interleukin
LDL	low-density lipoprotein
LE	lupus erythematosus
LKB1	liver kinase B1
LOX1	lectin-like oxidized LDL receptor
MACE	major adverse cardiovascular event
MAPK	mitogen-activated protein kinase
MCP1	monocyte chemoattractant protein 1
miRNA	micro ribonucleic acid
MMP-9	matrix metalloproteinase-9
MPO	myeloperoxidase
NF-κB	nuclear factor-kappa B
NO	nitric oxide
P2Y1	purinergic receptor Y1
PC	plasmalogen
PD	Parkinson’s disease
PDZK1	adaptor protein Na(+)/H(+) exchange regulatory cofactor NHE-RF3
PGI_2_	prostacyclin
PI3K	phosphatidylinositol 3-kinase
PKC®II	protein kinase C®II
PON1	paraoxonase 1
PSPB	pulmonary surfactant protein B (PSPB)
RA	rheumatoid arthritis
RCT	reverse cholesterol transport
rHDL	reconstituted high-density lipoprotein
ROS	reactive oxygen species
S1P	sphingosin-1-phosphate
S1PRs	sphingosin-1-phosphate receptors (1, 2 or 3)
SARS-CoV2	severe acute respiratory syndrome coronavirus 2
SAA	serum amyloid A
SDMA	symmetric dimethylarginine
SM	sphingomyelin
SR-BI (gene *SCARB1)*	scavenger receptor-BI
siRNA	small interfering ribonucleic acid
T2DM	type 2 diabetes mellitus
TLR-2	toll like receptor-2
TNF〈	tumor necrosis factor alpha
VCAM1	vascular cellular adhesion molecule 1
VEGF	vascular endothelial growth factor
VEGFR2	vascular endothelial growth factor receptor 2
